# Camouflage and interception: how pathogens evade detection by intracellular nucleic acid sensors

**DOI:** 10.1111/imm.13030

**Published:** 2018-12-18

**Authors:** Leonie Unterholzner, Jessica F. Almine

**Affiliations:** ^1^ Division of Biomedical and Life Sciences Faculty of Health and Medicine Lancaster University Lancaster UK

**Keywords:** DNA sensing, immune evasion, interferon, intracellular pathogens, retinoic‐acid inducible gene I‐like receptors, viruses

## Abstract

Intracellular DNA and RNA sensors play a vital part in the innate immune response to viruses and other intracellular pathogens, causing the secretion of type I interferons, cytokines and chemokines from infected cells. Pathogen RNA can be detected by retinoic‐acid inducible gene I‐like receptors in the cytosol, whereas cytosolic DNA is recognized by DNA sensors such as cyclic GMP‐AMP synthase (cGAS). The resulting local immune response, which is initiated within hours of infection, is able to eliminate many pathogens before they are able to establish an infection in the host. For this reason, all viruses, and some intracellular bacteria and protozoa, need to evade detection by nucleic acid sensors. Immune evasion strategies include the sequestration and modification of nucleic acids, and the inhibition or degradation of host factors involved in innate immune signalling. Large DNA viruses, such as herpesviruses, often use multiple viral proteins to inhibit signalling cascades at several different points; for instance herpes simplex virus 1 targets both DNA sensors cGAS and interferon‐*γ*‐inducible protein 16, as well as the adaptor protein STING (stimulator of interferon genes) and other signalling factors in the pathway. Viruses with a small genome encode only a few immunomodulatory proteins, but these are often multifunctional, such as the NS1 protein from influenza A virus, which inhibits RNA sensing in multiple ways. Intracellular bacteria and protozoa can also be detected by nucleic acid sensors. However, as the type I interferon response is not always beneficial for the host under these circumstances, some bacteria subvert, rather than evade, these signalling cascades for their own gain.

## Introduction

Our innate immune system provides a rapid, if relatively non‐specific, response to invading pathogens. The cornerstones of innate immunity are the pattern recognition receptors (PRRs), which detect pathogen‐associated molecular patterns (PAMPs) during infection. PAMPs are key molecular features shared by classes of pathogens that are usually absent from healthy cells, and include, for instance, bacterial or fungal cell wall components, or viral nucleic acids that would not usually be found in the host cell. PAMPs can be recognized *in trans* when phagocytes detect engulfed pathogens or debris from infected cells via Toll‐like receptors on their cell surface or inside endosomes. In addition, many cell types can also recognize when they have been infected themselves by an intracellular pathogen using intracellular PRRs that constantly survey the cell's contents for signs of infection. Intracellular PRRs raise the alarm either after sensing the presence of PAMPs directly, or after detecting the indirect effects of infection or tissue damage as danger‐associated molecular patterns.^1^

Intracellular nucleic acid sensors are PRRs that can perform both those functions: they can recognize pathogen‐derived DNA or RNA species inside the cell, and also detect nucleic acids as danger signals, for instance when infection leads to the leakage of mitochondrial DNA or RNA into the cytosol. Intracellular nucleic acid receptors are particularly important in the detection of viruses, which lack many other conspicuous features such as the unique cell wall components of bacteria or protozoa. However, nucleic acids are starting to emerge as key mediators during the innate immune response to a range of pathogens, including intracellular bacteria and protozoa.

In this review, we will focus on the intracellular DNA and RNA sensors that induce a transcriptional response leading to the production of interferons, cytokines and chemokines. The rapidly inducible transcriptional programme is a key factor in the early elimination of pathogens and for the initiation of appropriate local immune responses that ultimately determine the outcome of an infection. For this reason, it can be assumed that all pathogens need to evade or subvert the host's innate immune responses to be able to establish an infection. Here, we will describe the strategies that intracellular pathogens use to hide from the cell's nucleic acid receptors, and to disable or subvert their function. Ultimately, the activation of signalling cascades by nucleic acid sensors and many other PRRs converges on the activation of transcription factors such as nuclear factor‐*κ*B (NF‐κB) and interferon regulatory factor 3 (IRF3). Many pathogens also block innate immune responses at the level of these downstream signalling nodes, or by inhibiting the function of interferons and cytokines after they have been secreted, and we refer the reader to further reviews on this topic.^2^, ^3^, ^4^

## Detection of pathogens by RIG‐I‐like receptors

The RIG‐I‐like receptors (RLRs) retinoic‐acid inducible gene I (RIG‐I) and melanoma differentiation‐associated protein 5 (MDA5) recognize exogenous RNA in the cytosol of infected cells. RIG‐I detects short stretches of dsRNA as well as RNA species containing a triphosphate or diphosphate moiety at their 5′ end, and MDA5 recognizes long dsRNA molecules and higher‐order aggregates of viral dsRNA.^5^ Although the cytosol contains abundant cellular RNA molecules, these are not detected by RLRs. Messenger RNAs contain an m7‐guanosine cap at their 5′ end, tRNAs are cleaved to generate a 5′ monophosphate, and both tRNAs and rRNAs contain modifications that prevent recognition by RIG‐I.^5^ Double‐stranded RNA regions, e.g. in stem loops of endogenous RNA molecules, are usually short and contain frequent mismatches, further enhanced by the conversion of adenosine to inosine (A→I editing) mediated by adenosine deaminase acting on RNA 1 (ADAR1).^6^ In this way, RLRs are able to detect exogenous RNA molecules that may be present during infection in the presence of an abundance of cellular RNA in the cytosol.

Following detection of specific RNA ligands in the cytosol, RIG‐I and MDA5 undergo post‐translational modifications such as dephosphorylation and polyubiquitylation^7^ and signal via their caspase activation and recruitment domains (CARD) to the CARD‐containing adaptor mitochondrial anti‐viral signalling protein (MAVS), nucleating the assembly of prion‐like MAVS filaments.^8^ This results in the MAVS‐mediated activation of the transcription factors IRF3 and NF‐*κ*B, which co‐operate in the activation of the interferon‐*β* promoter. RLR signalling is further regulated by the addition of K48‐linked and K11‐linked ubiquitin chains, which mediate the degradation of RIG‐I and MAVS.^7^, ^9^ Termination of RLR signalling is also achieved by RIG‐I phosphorylation preventing the ubiquitination of CARDs^10^ and MAVS dephosphorylation, which impedes interaction with downstream signalling proteins.^11^ To prevent spurious activity, RIG‐I and MAVS exist in an autoinhibitory state facilitated by the phosphorylation and sequestration of CARDs in RIG‐I^12^, ^13^ and the repression of signalling motifs by adjacent regions in MAVS.^14^ Negative regulation of signalling is important to ensure that an innate immune response is transient, and does not cause extensive tissue damage.

The RLRs have been implicated in the detection of many viruses, as well as other intracellular pathogens. Most viral RNA polymerases generate transcripts with 5′ triphosphate ends and many viruses also generate dsRNA during their life cycle. Hence, both RIG‐I and MDA5 are predicted to be able to detect the dsRNA genomes of reoviruses and the dsRNA replicative intermediates of the positive‐strand ssRNA viruses of the *Flaviviridae* and *Coronaviridae* families.^15^ RIG‐I can also detect the short dsRNA panhandle structures containing a 5′ triphosphate from negative‐strand RNA viruses, such as influenza A virus (IAV) and Sendai virus,^16^, ^17^ and RNA with 5′ diphosphate ends, such as the genome of reoviruses.^18^ MDA5, on the other hand, is specifically required for the detection of picornaviruses, that contain protein‐capped positive‐stranded RNA genomes, and generate long, structured dsRNA intermediates that can be detected by MDA5, but not RIG‐I.^19^, ^20^, ^21^, ^22^ MDA5 also specifically detects ssRNA viruses of the *Caliciviridae* family, such as norovirus.^23^, ^24^ Several DNA viruses are also sensed by the RLRs, including herpes simplex virus 1 (HSV‐1), adenovirus, modified vaccinia virus Ankara and Epstein–Barr virus (EBV).^25^, ^26^, ^27^, ^28^, ^29^ This is thought to be the result of the convergent transcription of adjacent open reading frames generating dsRNA, and the transcription of structured RNAs by viral polymerases. RNA sensing can also contribute to the detection of intracellular bacteria, including *Legionella pneumophila*,* Salmonella typhimurium* and *Shigella flexneri*.^30^, ^31^, ^32^ Furthermore, RNA sensors can also be activated by cellular RNA as a positive feedback loop during infection: RNA fragments generated by the interferon‐stimulated gene, RNaseL, unmasked ribosomal RNA transcripts or mitochondrial RNA that leaks into the cytosol can also be detected by RLRs.^25^, ^33^, ^34^

## Detection of exogenous DNA and cyclic dinucleotides

Many mammalian cell types can also detect exogenous DNA. Bacterial, viral or synthetic dsDNA, and even dsDNA isolated from mammalian cells, can be sensed, if it gains access to the cytosol and is over 40–50 bp in length.^5^ The key sensor of cytosolic DNA that induces an interferon response is the enzyme cyclic GMP‐AMP synthase (cGAS), which upon dsDNA binding catalyses the production of the second messenger 2′3′‐cyclic‐GMP‐AMP (cGAMP).^35^ cGAMP then binds to the adaptor protein stimulator of interferon genes (STING), causing a conformational change in the STING dimers.^36^ This is linked to the activation of STING, its translocation from the endoplasmic reticulum to perinuclear signalling foci, and the association with TANK‐binding kinase 1 (TBK1) and IRF3.^36^, ^37^, ^38^ TBK1 phosphorylates STING at serine 366, which, in analogy to MAVS signalling, leads to the recruitment of IRF3.^37^ STING function is further regulated by palmitoylation, and modification with K63‐, K48‐, K11‐ and K27‐linked ubiquitin chains, and degradation by autophagy.^7^

Additional DNA binding proteins have been proposed to function as DNA sensors.^39^ As cGAS is essential for the response to cytosolic DNA in most systems investigated so far, any other proposed DNA sensors may synergise with cGAS as co‐receptors or co‐factors, in order to further amplify the response or to provide an additional layer of specificity. This has been shown for the DNA binding protein interferon‐*γ*‐inducible protein 16 (IFI16), which co‐operates with cGAS in the activation of STING in human cells.^40^, ^41^ In human cells, IFI16 may also be able to detect viral DNA in the nucleus, possibly by recognizing unchromatinized (‘naked’) stretches of DNA to distinguish viral DNA from our own chromatin.^42^, ^43^, ^44^ Another member of the PYHIN family, Absent in Melanoma 2 (AIM2), has also been shown to detect DNA.^45^, ^46^, ^47^ However, its downstream signalling diverges from IFI16 as it triggers inflammasome formation and interleukin‐1*β* production. Other DNA sensors include DNA‐dependent activator of interferon‐regulatory factors (DAI),^48^ DNA‐dependent protein kinase (DNA‐PK)^49^ DExD/H‐box helicases DHX9 and DHX36,^50^ DDX41^51^ and the double strand break repair endonuclease MRE11.^52^ Several of these proteins have been observed to be important for the induction of an interferon response after detection of cytosolic DNA, but their molecular role in the context of the cGAS–STING signalling axis remains to be determined.

The cytosolic DNA sensing pathway has been shown to be crucial for the detection of many different DNA viruses and retroviruses.^53^, ^54^ Surprisingly, some RNA viruses such as Dengue virus also engage the DNA sensing machinery by causing mitochondrial damage, resulting in the release of mitochondrial DNA into the cytosol.^55^ STING‐dependent DNA sensing has also been shown to be involved in the detection of DNA from protozoa such as *Plasmodium falciparum* and intracellular bacteria including *Mycobacterium tuberculosis* and *Listeria monocytogenes*.^56^, ^57^, ^58^, ^59^

During bacterial infection, the DNA sensing adaptor STING has a second function and can act as a PRR in its own right. STING can sense bacterial signalling molecules such as cyclic di‐GMP and cyclic di‐AMP.^60^, ^61^ Like cGAMP, these cyclic dinucleotides can bind and activate STING and induce the production of type I interferons, which has been observed, for example, during infection with *Mycobacterium tuberculosis* and *Listeria monocytogenes*.^61^, ^62^, ^63^ Hence bacteria can activate a STING‐dependent innate immune response by two different means, even though the resulting production of type I interferons is not always beneficial for the host, but instead can serve as an immune diversion strategy for some bacteria.^63^, ^64^

## Immune evasion strategies employed by intracellular pathogens

Pathogens and their hosts co‐evolve, and there are clear signs of selective pressure on host factors such as DNA and RNA sensors on the one hand and pathogen‐encoded immune evasion proteins on the other.^65^, ^66^, ^67^ Immunomodulatory proteins and virulence factors are finely tuned to adapt to the host range and life cycle of the pathogen. The precise interplay between host and pathogen factors is often unique, and can differ in different host organisms, cell types and among even closely related pathogens. However, mapping these host–pathogen interactions at a molecular level can provide clues about the importance of host signalling cascades, can help us to understand changes in species specificity and virulence of emerging pathogens, and aid in the design of vaccine vectors. As PRR signalling pathways are being characterized in more molecular detail, the intricate counterstrategies employed by pathogens to evade recognition are also becoming apparent. Some general principles by which intracellular pathogens hide from detection or intercept the cell's signalling cascades are described below.

### Sequestration of PAMPs

Pathogens that reside inside host cells are already sheltered from the immune system to some extent, by avoiding exposure to antibodies or complement components. However, due to the existence of intracellular PRRs, additional camouflage strategies are essential for the establishment of infection. Intracellular bacteria and protozoan parasites often co‐opt vacuoles as a niche for replication, which prevents pathogen‐derived nucleic acids and other PAMPs from being exposed to the cell's PRRs. Hence, detection of intracellular nucleic acids during bacterial infection often requires the presence of bacterial secretion systems. For instance, the type VII secretion system ESX from *Mycobacterium tuberculosis* is responsible for the exposure of bacterial DNA to cGAS in macrophages, but also secretes virulence factors that interfere with host functions.^56^, ^68^ Viruses require greater access to host factors for replication than bacteria, but also try to shield their nucleic acids from detection. Some RNA viruses, including Dengue virus and hepatitis C virus, sequester their genome and replication machinery in membrane‐bound compartments, which function to create locally high concentrations of replication factors, as well as hiding viral RNA genomes from recognition by RIG‐I.^69^, ^70^ Human immunodeficiency virus type 1 carries out reverse transcription and replication of its genome inside its capsid, which contains selective pores to allow entry for nucleotides from the host cell, while shielding viral DNA from recognition by cGAS.^71^

### Modification of viral RNA and DNA

Many pathogens aim to keep PAMP levels at a minimum: coronaviruses for instance quickly degrade any excessive dsRNA formed during the viral life cycle,^72^ and Group B streptococcus degrades cyclic di‐AMP using an ectonuclease.^73^ However, the production of nucleic acid PAMPs cannot be avoided altogether; thus, more sophisticated strategies of camouflage have evolved, so that the pathogen can blend into the cellular environment.

As viral RNA polymerases generate RNA species with a 5′ triphosphate moiety, many viruses employ additional strategies to modify their RNA, so that it is not recognized efficiently by RIG‐I. For instance, *Picornaviridae*,* Caliciviridae* and *Astroviridae* covalently attach a protein, Vpg, to the 5′ end of viral RNAs, which prevents RIG‐I‐mediated recognition.^15^ Processing of the negative‐strand ssRNA genome of members of the *Bornaviridae* and *Bunyaviridae* families involves the cleavage of the first nucleotide by a viral endonuclease to generate a monophosphate group at the 5′ end.^74^ Viruses that transcribe their mRNAs in the nucleus, including the DNA viruses of the *Herpesviridae* and *Papillomaviridae* families and retroviruses such as human immunodeficiency virus, co‐opt the cell's own capping machinery to protect the 5′ end of their mRNAs with a 7‐methylguanosine cap, while others, including IAV, use ‘cap snatching’ to remove the capped 5′ ends from cellular mRNAs and incorporate them into viral RNA transcripts.^75^ Many viruses, including poxviruses and flaviviruses, encode their own capping enzymes that synthesize RNA caps that are indistinguishable from the mammalian cap structure.^75^ Yellow fever virus in addition recapitulates the 2′ *O*‐methylation of the first RNA nucleotide next to the cap, which is found in cellular mRNAs and further decreases recognition by RIG‐I.^76^ Bacteria and protozoa also cap their mRNAs, but not always with 100% efficiency. RNA derived from intracellular bacteria can activate the RNA sensing pathway if it reaches the cytosol, but it remains to be determined whether this is important during infection.^77^

So far, it is not known whether DNA modifications could prevent recognition by cGAS or other DNA sensors. It has been proposed that IFI16 may be able to distinguish viral DNA from the cell's own genome by recognizing longer (>40 nucleotides) stretches of free DNA,^43^, ^44^ and the detection of DNA by cGAS has also been shown to be length‐dependent.^78^ It remains to be tested whether the assembly of nucleosomes on the genomes of DNA viruses such as herpesviruses and papillomaviruses could be a strategy to limit their detection by intracellular DNA sensors.

### Degradation and inhibition of host signalling factors

All intracellular pathogens encode virulence factors that interfere with the host's signalling cascades. Viral immunomodulatory proteins are usually expressed as immediate early genes, or are even part of the viral particle, so that they are able to inhibit the host's signalling cascades as soon as PAMPs are detected early in infection. Intracellular bacteria employ secretion systems to transport effector proteins through the vacuolar and bacterial membranes into the host cell, whereas protozoans can secrete virulence factors via exosomes or other membrane‐bound vesicles. A vast repertoire of virulence factors that influence the cell's innate immune signalling cascades have been described, particularly in viruses, which often dedicate a large portion of their genome to interfering with the host's innate immune response. Although many viruses encode only a few proteins, these are often multi‐functional and target several key host factors. Large DNA viruses, such as poxviruses and herpesviruses, encode a larger repertoire of dozens of immunomodulatory proteins that interfere with anti‐viral signalling cascades in the cell at multiple points.^79^, ^80^ Bacteria and protozoa encode thousands of genes, and so have an even greater capacity to interfere with host functions, but comparatively few immune evasion proteins have been described so far, with many pathogen‐encoded effector proteins still uncharacterized.^3^, ^4^, ^64^

Many pathogens have evolved similar strategies to inhibit the innate immune signalling cascades in the host cell: virulence factors act to either eliminate host signalling factors by degradation, sequester them or block their function in the signalling cascade. As we learn more about the regulation of innate immune signalling cascades that detect pathogens, more examples of counterstrategies employed by pathogens also emerge.

#### Degradation and inhibition of RNA sensors

**Figure 1:**
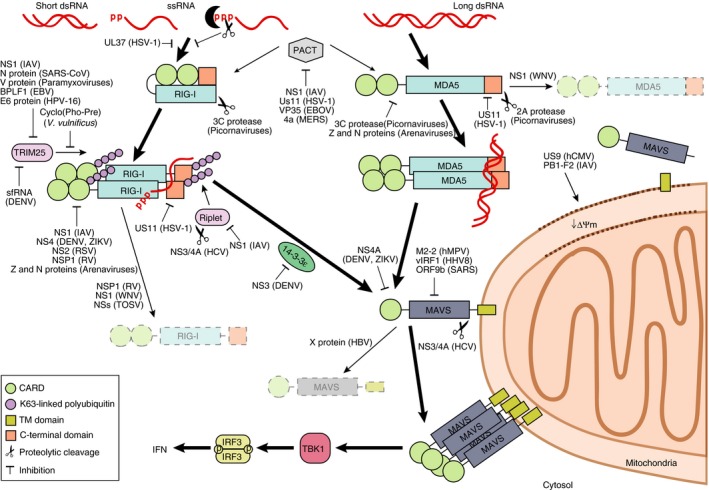
Schematic representation of the pathogen‐derived molecules employed to evade the intracellular RNA sensing pathway. The RNA sensors RIG‐I and MDA5 detect various RNA species, primarily those containing 5′ di‐ or tri‐phosphates or long dsRNA, respectively. Some viruses modify or mask their viral RNA, for instance by cleaving 5′ phosphates, attaching a viral protein or ‘cap snatching’. Pathogens also inhibit RIG‐I activation via degradation or blocking accessory proteins (TRIM25 and Riplet), by direct binding with the CARD or C‐terminal domains of RIG‐I and by preventing RIG‐I translocation to the mitochondria to block its interaction with MAVS. MDA5 is targeted in a similar fashion, often by degradation or inhibition by binding of virulence factors. Various viral proteins also interact with or degrade MAVS, which provides a further layer of evasion of the RNA sensing pathway. DENV, Dengue virus; EBV, Epstein–Barr virus; EBOV, Ebola virus; HBV, hepatitis B virus; hCMV, human cytomegalovirus; HCV, hepatitis C virus; HHV8, human herpesvirus 8; hMPV, human metapneumovirus; HSV‐1, herpes simplex virus 1; IAV, influenza A virus; MERS, Middle Eastern respiratory virus; RSV, respiratory syncytial virus; RV, rotavirus; SARS, severe acute respiratory syndrome; TOSV, Toscana virus; WNV, West Nile virus; ZIKV, Zika virus.

Since the discovery of the intracellular RNA sensing system, many immune evasion proteins that target RLRs or MAVS have been identified (see Fig. 1), mostly in viruses with an RNA genome, for which RIG‐I and/or MDA5 are the key PRRs during infection. Some viral proteases cleave RNA sensors, with several picornaviruses using their 3C protease to cleave RIG‐I, and the 2A protease to cleave MDA5.^81^, ^82^, ^83^ The leader protease L^pro^ from foot‐and‐mouth‐disease virus also cleaves Lpg2, a co‐factor for MDA5 activation.^84^ Some viruses modify RLRs with K48‐linked poly‐ubiquitin chains, diverting the RNA sensors for degradation by the cell's proteasome. Toscana virus non‐structural proteins and the rotavirus NSP1 protein cause the degradation of RIG‐I,^85^, ^86^ whereas West Nile virus NS1 targets both RIG‐I and MDA5.^87^ RIG‐I mRNA expression and translation can also be inhibited during viral infection: for instance, EBV expresses the virus‐encoded microRNA miR‐BART6‐3p, which targets RIG‐I mRNA,^88^ and hepatitis B virus induces the cellular microRNA miR146a, which inhibits RIG‐I expression.^89^

Even under conditions where the expression of RIG‐I and MDA5 is intact, the RNA sensors can be prevented from carrying out their signalling function by viral proteins that bind to them. The HSV‐1 protein UL37 interacts with RIG‐I and causes the de‐amidation of its helicase domain, which renders RIG‐I unable to sense RNA ligands.^90^ Another HSV‐1 protein, US11 inhibits RNA sensing by associating with the dsRNA binding protein PACT, which potentiates RNA‐induced responses.^91^, ^92^ PACT is also targeted by Influenza A virus NS1, the VP35 protein from Ebola virus and the 4a protein from Middle East respiratory syndrome coronavirus.^93^, ^94^, ^95^ Many of the PACT‐interacting proteins also bind dsRNA, so they probably shield the viral RNA from detection while at the same time inactivating PACT and RNA sensors.

The activity of RIG‐I and MDA5 is tightly regulated in the cell: In the absence of infection, the CARD of the RNA sensors are kept in a phosphorylated state, and activation involves their dephosphorylation catalysed by protein phosphatase 1.^96^ RIG‐I activity is further enhanced by the assembly of K63‐linked poly‐ubiquitin chains catalysed by the E3 ubiquitin ligases tripartite motif containing 25 (TRIM25) and Riplet, which release RIG‐I from its autoinhibited state.^97^, ^98^ Given the importance of these post‐translational modifications, it is not surprising that many viruses have evolved ways of inhibiting these regulatory mechanisms. For instance, measles virus uses an elaborate signalling strategy involving the activation of the C‐type lectin DC‐SIGN to inhibit protein phosphatase 1, thus keeping the RLRs phosphorylated and inactive.^99^ The E3 ubiquitin ligase Riplet is inhibited by the multifunctional IAV NS1 protein^100^ and is cleaved by the NS3/4A protease from hepatitis C virus.^101^ Multiple viruses also inhibit TRIM25, including again IAV NS1, the SARS coronavirus N protein, the V protein from various paramyxoviruses, the large tegument protein BPLF1 from EBV and the E6 protein from human papillomavirus 16.^102^, ^103^, ^104^, ^105^, ^106^, ^107^ The subgenomic flavivirus RNA from Dengue virus can also bind and inactivate TRIM25 in an RNA sequence‐specific manner.^108^ RIG‐I ubiquitylation by TRIM25 is also inhibited by the bacterial quorum sensing molecule cyclo(Phe‐Pro) from the opportunistic pathogen *Vibrio vulnificus*. Cyclo(Phe‐Pro) specifically binds to RIG‐I and prevents it from being ubiquitylated by TRIM25,^109^ possibly providing a link between bacterial infection and susceptibility to co‐infection with viruses.

A multitude of viral immunomodulators prevent RIG‐I and MDA5 from interacting with its adaptor protein MAVS, so blocking the nucleation event that allows MAVS to form higher‐order signalling assemblies. Dengue virus NS3 binds 14‐3‐3*ε* to prevent the translocation of RIG‐I to MAVS.^110^ The interaction between RLRs and MAVS is also disrupted by the picornavirus 3C^pro^ proteases, the Z and N proteins of some arenaviruses, NS2 from respiratory syncytial virus, NS1 from IAV, NSP1 from rotavirus and US11 from HSV‐1.^16^, ^86^, ^111^, ^112^, ^113^, ^114^

MAVS is also targeted directly by many viruses. MAVS‐interacting proteins include the metapneumovirus protein M2‐2,^115^ the NS4A proteins of Zika and Dengue viruses,^116^, ^117^ viral IRF1 from human herpesvirus 8^118^ and ORF9b from SARS coronavirus.^119^ MAVS is also diverted for proteasome‐mediated degradation by the hepatitis B virus X protein,^120^ and is cleaved by the hepatitis C virus protease NS3/4A, which releases it from the mitochondria and other intracellular membranes to disrupt signalling.^121^, ^122^, ^123^, ^124^ The US9 protein from HSV‐1 causes MAVS to leak from the mitochondria by disrupting the mitochondrial membrane potential,^125^ and the IAV protein PB1‐F2 decreases mitochondrial membrane potential causing mitochondrial fragmentation.^126^, ^127^ Disrupting mitochondrial physiology is a common mechanism to block MAVS signalling. Infection with Dengue and Zika viruses causes mitochondrial elongation, which also blocks MAVS function.^128^ The sustained interferon production caused by MAVS signalling from the peroxisomes is inhibited by the MIA protein from human cytomegalovirus (hCMV) and the VP16 protein from HSV‐1.^118^, ^129^, ^130^

Although there are abundant examples of inhibition of RNA sensing by pathogens, and in particular viruses, there is one instance where a pathogen actually enhances signalling by RIG‐I and MDA5. The food‐borne pathogen *Salmonella typhimurium* uses its effector protein SopA, an E3 ubiquitin ligase, to ubiquitylate the host proteins TRIM56 and TRIM65, which in turn promote RIG‐I and MDA5 signalling.^131^, ^132^ This is an example of subversion, rather than evasion, of the interferon response, in line with the observation that the production of type I interferons can be advantageous to the pathogen, rather than the host, during some bacterial infections.^64^

#### Degradation and inhibition of DNA sensors

**Figure 2:**
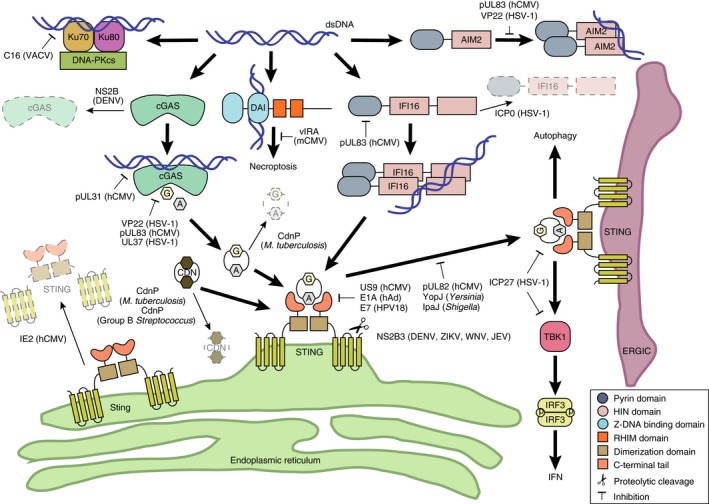
Schematic representation of the pathogen‐derived molecules employed to evade the intracellular DNA sensing pathway. cGAS is a sensor of cytosolic DNA and the main activator of the adaptor protein, STING. Pathogens target cGAS for degradation, impede cGAS from binding DNA or inhibit its catalytic activity. Pathogens also degrade cGAMP, the second messenger produced by cGAS, as well as bacterial cyclic dinucleotides. IFI16 contributes to the activation of the cGAS–STING pathway, and several viral proteins have been identified that inhibit its activation and promote its degradation. Other DNA sensors, such as DNA‐PK, DAI and AIM2 are also targeted by viral factors that block DNA binding and activation of downstream pathways. Pathogens also hinder multiple aspects of STING function through proteolytic cleavage, degradation, blocking its translocation from the endoplasmic reticulum (ER) to ER‐Golgi intermediate compartment (ERGIC) and preventing interaction with downstream signalling proteins, such as TANK‐binding kinase 1 (TBK1). DENV, Dengue virus; hAd, human adenovirus; hCMV, human cytomegalovirus; HSV‐1, herpes simplex virus 1; HPV18, human papillomavirus 18; JEV, Japanese encephalitis virus; mCMV, mouse cytomegalovirus; WNV, West Nile virus; ZIKV, Zika virus.

Even though the cGAS/STING DNA sensing pathway was only discovered relatively recently, several viral and bacterial virulence factors that target this pathway have already been discovered, see Fig. 2. The nuclear DNA viruses hCMV, HSV‐1 and Kaposi's sarcoma‐associated herpesvirus all target several key DNA sensing factors to prevent the detection of their genomic DNA. HSV‐1 uses its ICP27 protein to inhibit STING.^133^ In addition, the E3 ubiquitin ligase ICP0 causes the degradation of IFI16 protein, and the virion host shutoff protein UL41 promotes the turnover of the mRNAs encoding IFI16 and cGAS.^42^, ^134^, ^135^ In addition, the HSV‐1 virion protein VP22 interacts with cGAS and inhibits its enzymatic activity^136^ and also interacts with AIM2 and blocks its oligomerization.^137^ HSV‐1 tegument protein UL37, which targets and deamidates RIG‐I, also deamidates cGAS, impairing cGAMP synthesis.^138^ The deployment of different virulence factors to target several components of the DNA sensing pathway may be necessary to block the pathway more efficiently, and/or to inhibit some non‐overlapping functions of cGAS and IFI16 which have been observed during herpesvirus infection.^139^ Analogously, several different hCMV proteins inhibit DNA sensing: pUL31 binds to cGAS and dissociates it from DNA,^140^ pUL83 inhibits both cGAS and IFI16,^141^, ^142^, ^143^ pUL82 inhibits STING translocation,^144^ IE2 causes STING degradation,^145^ and US9 inactivates both MAVS and STING.^125^ Furthermore, pUL83 binds AIM2 and facilitates the degradation of AIM2‐driven inflammasomes.^146^ A similar multi‐pronged approach has also been observed for the gammaherpesvirus Kaposi's sarcoma‐associated herpesvirus.^147^, ^148^, ^149^, ^150^ STING is also inhibited by oncoproteins from the nuclear DNA viruses human adenovirus and human papillomavirus,^151^ and by vaccinia virus, a DNA virus that resides in the cytosol.^152^ Vaccinia virus also inhibits DNA‐PK, using its virulence factor C16 to bind the Ku70/Ku80 subunits of the DNA‐PK complex.^153^ The viral inhibitor of RIP activation (vIRA) from mouse CMV targets another proposed DNA sensor, DAI, to prevent the induction of programmed necrosis upon viral infection.^154^

Surprisingly, not only DNA viruses but also some RNA viruses have been found to antagonise STING – even though they do not produce DNA or cyclic dinucleotides during their replication cycle. It has been reported that STING can function in a cGAS‐independent manner during the detection of viral membrane fusion, for instance during infection with IAV.^155^ This non‐canonical STING signalling pathway is antagonized by the IAV fusion peptide.^156^ Flaviviruses, enveloped viruses with a positive‐stranded ssRNA genome, also inhibit STING‐dependent DNA sensing. Viral NS2B proteases encoded by Dengue virus, Zika virus, West Nile virus and Japanese encephalitis virus cleave human STING protein, but not its mouse orthologue.^157^, ^158^ The dengue virus NS2B co‐factor also targets cGAS for degradation.^55^ This prevents the activation of an innate immune response after the release of mitochondrial DNA, which occurs during Dengue virus infection,^55^, ^159^ highlighting the breadth of pathogen classes that can be detected directly or indirectly by the DNA sensing pathway (Fig. 2).

While the production of type I interferons is not always an effective strategy to limit bacterial infections, the activation of STING by bacterial DNA or cyclic dinucleotides can also promote autophagy to clear bacteria from the infected cells. For this reason, some intracellular bacteria also inhibit STING.^62^ For instance, the *Shigella* effector protein IpaJ inhibits STING translocation from the endoplasmic reticulum to endoplasmic reticulum–Golgi intermediate compartment,^38^ and the *Yersinia* YopJ protein also blocks STING trafficking and causes its de‐ubiquitylation.^160^ *Mycobacterium tuberculosis* secretes the cyclic di‐nucleotide phosphodiesterase CdnP (also known as Rv2837c), which degrades cGAMP and bacterial cyclic dinucleotides, thus inhibiting both DNA‐ and cyclic‐dinucleotide‐induced STING activation.^161^ Group A streptococcus, *Streptococcus pyogenes*, subverts, rather than evades, STING signalling: it uses its M protein to activate STING, resulting in the production of the anti‐inflammatory cytokine interleukin‐10 downstream of type I interferon signalling.^162^ In this way, the bacterium exploits the reciprocal antagonism between the interferon response and inflammation, and shapes the immune response to favour the pathogen, rather than the host.

## Concluding remarks

All pathogens, having co‐evolved with their hosts, employ elaborate strategies to strike a fine balance between the requirements of their own life cycle, the evasion of host defences, and effects on pathology for the host, which can influence further transmission and hence the evolutionary fitness of a pathogen. As in so many fields in biology, much can be learnt from insights into the host–pathogen interactions that interfere with our cells' defences. Immunomodulatory proteins from pathogens can point us to their targets, which are often key signalling nodes that play a role during infection. Although immune evasion has long been studied in human cells and mouse models, the investigation of innate immune signalling in reservoir hosts such as bats or birds, is an emerging field of research. The variation of these interactions during pathogen evolution often underlies the ability of pathogens to cross species boundaries or to adopt new traits in transmission or virulence during emerging infections. Knowing more about the functions of individual immunomodulatory proteins is also crucial for the rational development of vaccine vectors, as for instance the removal of individual immune evasion factors can aid safety and immunogenicity of a live vaccine. It is becoming increasingly clear that nucleic acid PAMPs and their PRRs also perform key roles during sterile inflammation, autoimmunity and even cancer treatment, so the lessons we learn from our pathogens may ultimately have applications for the treatment and prevention of both infectious and non‐infectious diseases that involve the immune system.

## Disclosures

The authors declare no competing interests.
